# Early cleft lip repair in an infant with cleft lip–palate and ipsilateral microtia identified through a community outreach program

**DOI:** 10.1093/jscr/rjag448

**Published:** 2026-06-10

**Authors:** Agus Santoso Budi, Kartika Ayu Pranindita

**Affiliations:** Department of Plastic, Aesthetic & Reconstructive Surgery, Faculty of Medicine, Universitas Airlangga/Dr. Soetomo Hospital, Jl. Prof. DR. Moestopo No. 6-8, Airlangga, Kec. Gubeng, Surabaya, Jawa Timur 60286, Indonesia; Faculty of Medicine, Public Health, and Nursing, Universitas Gadjah Mada, Jl. Farmako, Sendowo, Sekip Utara, Kec. Depok, Kabupaten Sleman, Daerah Istimewa Yogyakarta 55281, Indonesia

**Keywords:** cleft lip, cleft palate, microtia, craniofacial anomalies, outreach surgery

## Abstract

Cleft lip and palate are common craniofacial anomalies that may affect feeding, speech, and hearing. When associated with external ear malformations such as microtia, careful evaluation for auditory impairment and possible syndromic conditions is required. We report a 3-month-old male infant with a left complete unilateral cleft lip and palate and ipsilateral Weerda grade II microtia who underwent early cleft lip repair through an outreach cleft care program. The patient presented with feeding difficulty but no additional craniofacial or systemic anomalies. Surgical repair using a rotation-advancement technique was performed. This case highlights the importance of early recognition and referral through community networks to enable timely surgical management of craniofacial anomalies in resource-limited settings.

## Introduction

Cleft lip and palate are among the most common congenital craniofacial anomalies worldwide, affecting approximately 1 in 700 live births [1]. These conditions impact feeding, growth, hearing, speech development, and psychosocial well-being, requiring coordinated multidisciplinary management from infancy through adolescence [2]. When cleft occurs with external ear malformations such as microtia, airway, and auditory considerations become more prominent, and the possibility of syndromic associations must be evaluated [3].

Timely cleft lip repair improves feeding efficiency and supports early social interaction [4]. However, access to cleft specialists is limited to tertiary centres, leading to delays in care due to geographic and socioeconomic barriers. Outreach-based surgical programs provide a pathway for earlier intervention and help establish continuity of care in underserved settings [5].

This report describes a 3-month-old male infant with a left complete unilateral cleft lip, alveolus, hard, and soft palate associated with ipsilateral microtia who underwent early surgical management through an outreach service operating in an underserved region.

## Case report

A 3-month-old male infant with a visible left complete unilateral cleft lip and ipsilateral microtia was brought to a cleft outreach program in a rural district. The family resides in a remote village, where specialist cleft services are unavailable locally. Referral logistics were supported through collaboration between the local primary health center, a district hospital, and a non-profit organization focused on improving access to cleft care.

The infant was born at term by caesarean delivery with a birth weight of 3.7 kg from an uncomplicated pregnancy. Antenatal ultrasound examinations did not detect the cleft anomaly. The mother reported intermittent folic acid intake. There was no family history of congenital anomalies and no parental consanguinity.

Before surgery, the infant was bottle-fed due to difficulty in breastfeeding effectively. Feeding required additional time and the infant’s weight at presentation was 5 kg. Newborn hearing screening was not available, although caregivers reported normal startle responses.

**Figure 1:**
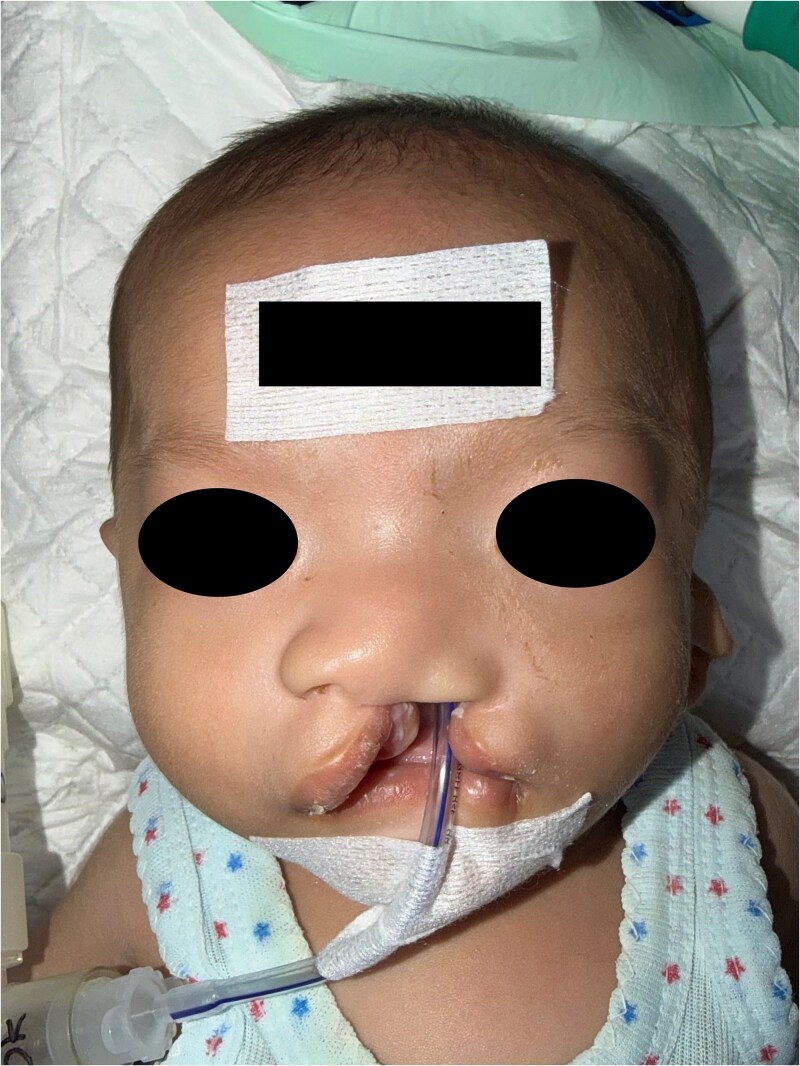
Preoperative frontal view showing a left complete unilateral cleft lip.

**Figure 2:**
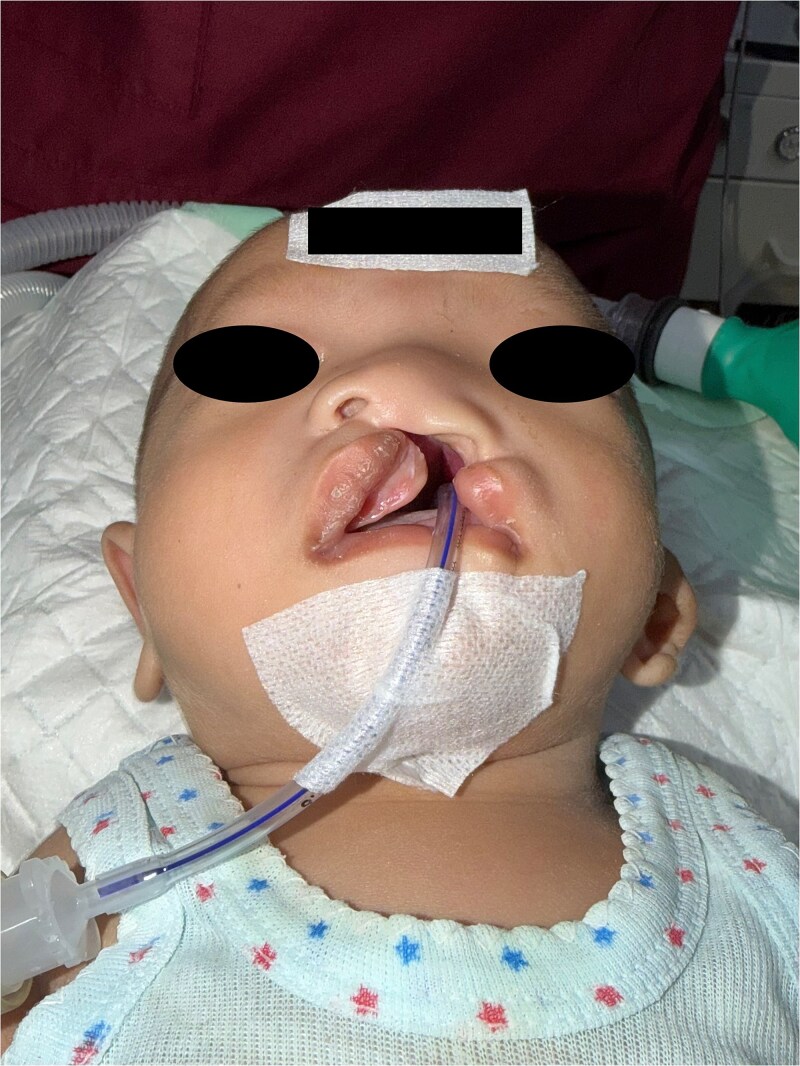
Basal view demonstrating the cleft deformity involving the lip and alveolus with nasal asymmetry.

**Figure 3:**
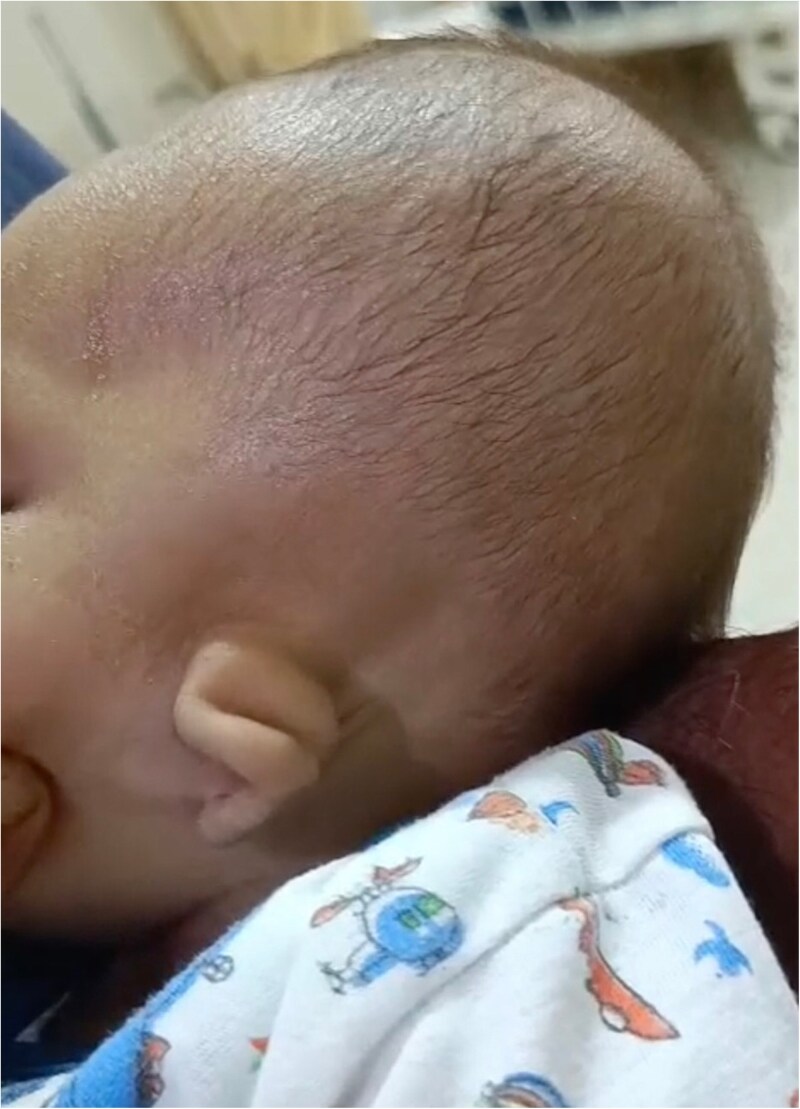
Left lateral view demonstrating Weerda grade II microtia.

**Figure 4:**
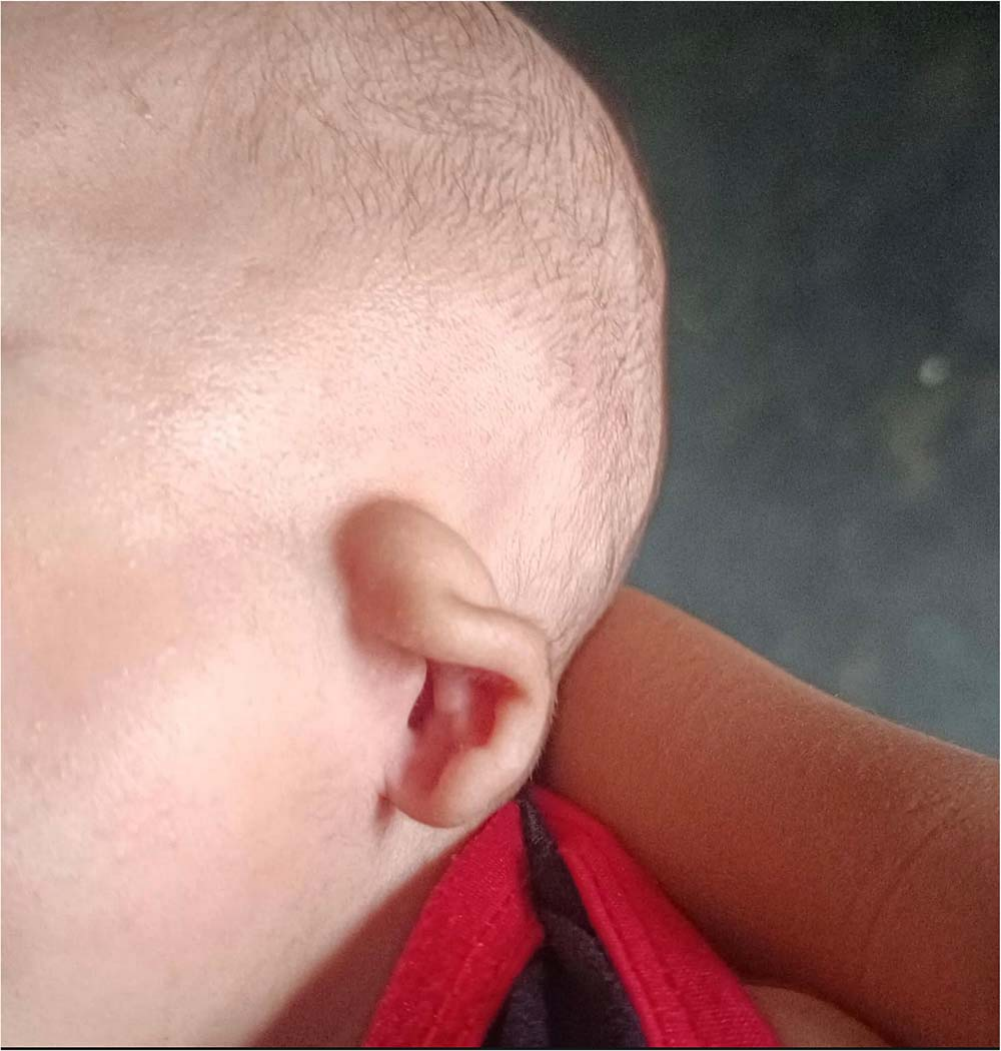
Close-up view of the left auricle demonstrating a patent external auditory canal.

Physical examination confirmed a left complete cleft lip and alveolus extending into the hard and soft palate (Fig. 1), classified as —SHAL in the LAHSHAL system and consistent with a Veau Class III cleft. Left alar collapse and nasal asymmetry were noted (Fig. 2). The left ear showed Weerda Grade II microtia (Fig. 3) with a narrow but patent external auditory canal (Fig. 4), while the right ear was normal.

**Figure 5:**
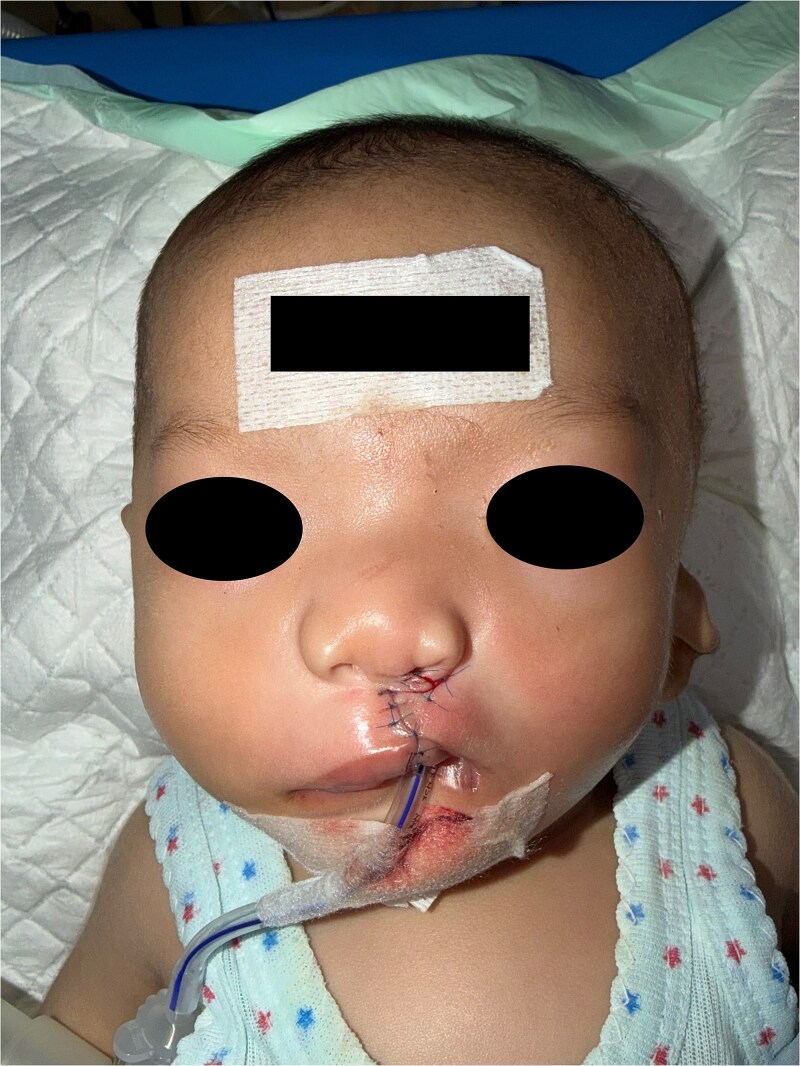
Early postoperative frontal view following unilateral cleft lip repair using a rotation-advancement technique.

**Figure 6:**
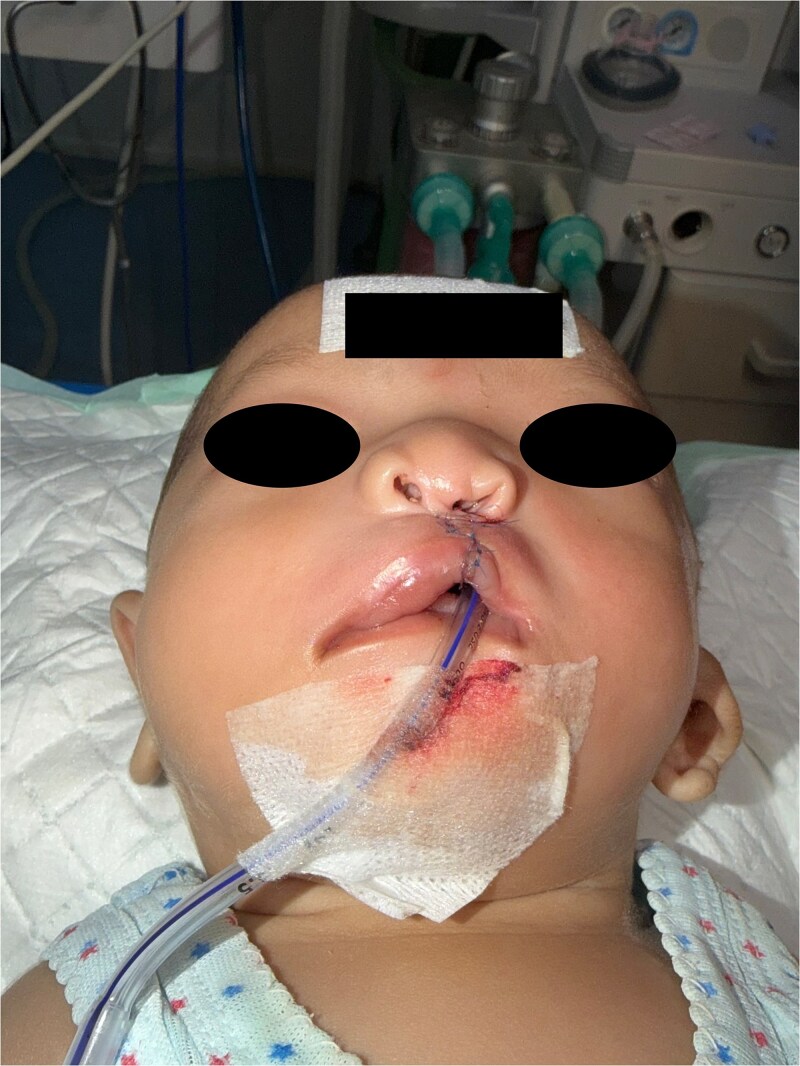
Early postoperative basal view demonstrating restoration of lip continuity and improved nasal symmetry.

Unilateral cleft lip repair was performed under general anaesthesia using the Millard rotation-advancement technique (Figs 5 and 6). Caregivers were advised on wound care and warning signs. Follow-up was arranged to monitor healing and feeding progress. The family received counselling regarding staged cleft care, including palatoplasty at 9–12 months of age, hearing evaluation when accessible, nutritional monitoring, and later consideration of auricular reconstruction.

## Discussion

This case describes unilateral cleft lip and palate occurring together with ipsilateral microtia, anomalies that may arise from shared developmental pathways. Microtia is a congenital malformation of the external ear with an estimated incidence of 1 in 6000–12 000 live births and occurs more frequently in males and in unilateral form [6]. Up to 20%–60% of affected children present with additional anomalies or syndromes, most commonly involving the craniofacial structures [3]. Population-based birth-defects surveillance studies reported that external ear anomalies frequently occur alongside other craniofacial malformations, including cleft lip and palate [7].

These associations reflect their shared embryologic origin. Craniofacial structures develop from cranial neural crest cells that migrate from the neural tube into the first and second pharyngeal arches during weeks 4–5 of embryogenesis. These cells contribute to the formation of facial prominences involved in lip and palate development, as well as the auricular hillocks of His that form the external ear. Disruption of neural crest cell migration or proliferation during this period may impair fusion of the maxillary prominence with the medial nasal process, resulting in cleft lip, while simultaneously affecting the auricular hillocks, producing microtia [8, 9]. This shared developmental origin likely explains the concurrent anomalies observed in the patient. Although cleft lip and palate may co-occur with external ear anomalies, reports describing complete unilateral cleft-lip palate with ipsilateral microtia and patent external auditory canal remain limited [10].

Even when the external auditory canal appears patent, children with microtia remain at risk of conductive or mixed hearing loss. Early multidisciplinary management and audiologic assessment are essential to detect hearing impairment and prevent potential delays in speech and language development [6, 11]. In this case, formal audiologic testing was not available and evaluation is planned during follow-up.

The coexistence of microtia and clefting should also prompt evaluation for a possible syndromic condition. Microtia is a key feature in several craniofacial syndromes, such as craniofacial microsomia, Townes–Brocks syndrome, and Treacher Collins syndrome, and is frequently seen within the Oculo-Auriculo-Vertebral Spectrum, which may involve facial asymmetry, ear or facial tags, epibulbar dermoid, and vertebral, renal, or cardiac anomalies [3, 6]. Hypertelorism–Microtia–Clefting syndrome, should also be considered [12]. In this patient, the absence of hypertelorism, facial asymmetry, or systemic abnormalities made a syndromic diagnosis less likely, although continued follow-up remains important.

Feeding difficulty and mild growth are common in infants with cleft lip and palate because the absence of an effective lip seal reduces feeding efficiency [13]. Early lip repair at 3 months helped restore lip continuity and improve oral competence, facilitating feeding before palatal repair.

From a health-systems perspective, this case illustrates how community-linked outreach programs can improve access to specialized craniofacial care in underserved populations. In many low- and middle-income countries, access to comprehensive cleft care remains limited, with long travel distances and financial constraints representing major barriers to treatment and follow-up. Outreach networks that connect community health workers, primary care facilities, district hospitals, and cleft-focused non-profit organizations can facilitate earlier detection and timely surgical intervention [14, 15]. In certain countries, where cleft teams remain concentrated in major urban centres, such referral pathways are essential to reduce treatment delays. In this case, early recognition by a community health volunteer enabled cleft lip repair at 3 months and entry into a structured pathway of staged cleft care [5].
